# Beyond the clinic: a six-month home-based exercise and respiratory rehabilitation program improves functional capacity, respiratory muscle strength, and fatigue in chronic heart failure

**DOI:** 10.3389/fresc.2026.1958027

**Published:** 2026-10-14

**Authors:** Mariusz Ciołkiewicz, Mariusz Wojciuk, Anna Kuryliszyn-Moskal, Agnieszka Dakowicz, Emilia Sawicka-Śmiarowska, Karol Kamiński

**Affiliations:** 1 Department of Rehabilitation, Medical University of Bialystok, Bialystok, Poland; 2 Department of Population Medicine and Lifestyle Diseases Prevention, Medical University of Bialystok, Bialystok, Poland; 3 Department of Cardiology and Internal Medicine with Cardiac Intensive Care Unit, Medical University of Bialystok, Bialystok, Poland

**Keywords:** cardiac rehabilitation, HFrEF, home-based exercise program, respiratory muscle strength, respiratory training

## Abstract

**Introduction:**

Optimal models of rehabilitation dedicated for patients with heart failure (HF) remain under investigation. The aim of this study was to assess the safety and effectiveness of a 6-month home-based rehabilitation program in patients with HF.

**Materials and methods:**

63 patients with stable HF were randomized, 28 patients from intervention group completed the study and were included in the analysis. Assessments including the 6-minute walk test, respiratory muscle strength, and quality of life measures (SF-36 and Fatigue Severity Scale – FSS) were conducted at baseline and after 6 months. Additionally, adverse events and adherence to the training protocol were monitored.

**Results:**

Due to objective challenges caused by the COVID-19 pandemic outbreak, the course and final outcomes of the study differed from the original protocol; the control group was not assessed after 6 months. In the intervention group, 6 months of rehabilitation resulted in statistically significant improvements: the 6-minute walk distance increased by 37.86 meters (*p* = 0.001), maximal inspiratory pressure (PImax) improved by 20.21 cmH₂O, and maximal expiratory pressure (PEmax) by 20.75 cmH₂O (both *p* < 0.01). A significant reduction in fatigue was observed, as measured by FSS, with a mean decrease of 0.68 points (*p* = 0.029). Adherence to the exercise protocol averaged 91.5 ± 15.3%. No serious adverse events were reported.

**Conclusions:**

The home-based rehabilitation program, including respiratory muscle training, is safe and effective for patients with chronic HF. It improves functional capacity and reduces fatigue levels.

**Clinical Trial registration:**

https://clinicaltrials.gov/ct2/show/NCT03780803, ClinicalTrials.gov, NCT03780803. Registered 19/12/2018.

## Introduction

Chronic heart failure (CHF) is associated not only with pulmonary congestion and altered gas exchange but also with impaired lung mechanics, increased work and cost of breathing (1, 2).

Exercise training (ET) improves physical endurance and quality of life, and reduces all-cause as well as heart failure–related hospitalizations (3–7). According to recent data, ET may also reduce all-cause mortality over the long term (8, 9). The beneficial effect on hospitalizations is particularly pronounced in patients who are highly adherent to the rehabilitation regimen (10).

Another important component of cardiac rehabilitation dedicated to CHF patients is respiratory training composed of breathing exercises (BE) and inspiratory muscle training (IMT). Respiratory training in CHF improves not only ventilation and ventilation-to-perfusion matching but also physical endurance and quality of life (11–14).

According to the actual European Society of Cardiology (ESC) guidelines, supervised exercise training and rehabilitation are recommended for all patients with CHF who are able to participate. An inpatient exercise-based cardiac rehabilitation program should also be considered for patients with more severe disease, frailty, or comorbidities (3).

There is a need to develop specialized improvement programs and to identify safe and effective training models in future studies. To date, only a small number of studies exploring home-based, unsupervised rehabilitation models for patients with CHF have been published, with relatively short observation periods and interventions mainly limited to exercise or walking. Available data suggest that home-based rehabilitation is non-inferior to centre-based rehabilitation in terms of safety and completion rates (15).

The aim of the present study was to evaluate the efficacy and safety of a 6-month home-based rehabilitation program, previously successfully tested in a group of patients with pulmonary arterial hypertension (16) on symptoms, physical endurance, respiratory and skeletal muscle strength, and quality of life in stable patients with heart failure with reduced ejection fraction (HFrEF).

## Materials and methods

### Study population

The prospective interventional pilot study was approved by the Bioethics Committee of the Medical University of Bialystok (R-I-002/17) and conducted in accordance with the Declaration of Helsinki. The study protocol was registered at ClinicalTrials.gov (NCT03780803). This study was conducted as part of a broader rehabilitation research program registered under ClinicalTrials.gov NCT03780803, which also included a previously published cohort with pulmonary arterial hypertension (16). The present analysis concerns the HFrEF arm of this registered program. Written informed consent was obtained from all patients prior to their inclusion. The study was conducted at the Rehabilitation Department of the Medical University of Bialystok, Poland, between October 2019 and July 2021. Participants were patients from the Department of Cardiology of the University Teaching Hospital in Bialystok and the Cardiology Outpatient Clinic, diagnosed with HFrEF according to ESC guidelines, with left ventricular ejection fraction <40%. All patients were classified as New York Heart Association (NYHA) class II or III and were clinically stable during the three months prior to the study. No significant modifications in CHF medical treatment or electrotherapy were made during the study period.

Exclusion criteria were: CHF in NYHA class IV; significant changes in CHF treatment, acute coronary syndrome or syncope within three months prior to the study; severe pulmonary diseases; advanced cancer; low hemoglobin levels (<11 g/dL for males, <10 g/dL for females); hormonal disorders, electrolyte imbalances, or acute inflammatory states within four weeks before enrolment; and other clinical conditions precluding participation in cardiac rehabilitation.

Patients were randomized to either the control or intervention group in a 3:2 ratio. Randomization was performed using Research Randomizer (Version 4.0).

Healthy persons with no cardiovascular, other significant chronic diseases or medical conditions potentially confounding clinical evaluation, constituted the reference group.

### Clinical assesment

All participants underwent clinical evaluation, including a full medical history, anthropometric measurements, and physical examination.

The primary outcome measure, the 6-minute walk test (6MWT), was performed according to the American Thoracic Society guidelines (17). Heart rate and blood pressure were measured, and dyspnoea and fatigue were assessed using the 10-point modified Borg Scale before and after the test. Blood oxygen saturation was continuously monitored throughout the test and recorded before walking, as well as at the second, fourth, and sixth minutes

Transthoracic echocardiography was performed using the iE33x Matrix device (Philips) according to the guidelines recommended by the American Society of Echocardiography (18). Left ventricular ejection fraction was calculated with the use of Simpson's biplane method.

Respiratory muscle strength was assessed using the MicroRPM Respiratory Pressure Meter (Micro Medical Ltd., Rochester, Kent, UK). Both maximum inspiratory pressure (PImax) and maximum expiratory pressure (PEmax), generated during forced inspiration and expiration, were measured according to the guidelines of the American Thoracic Society and European Respiratory Society (19, 20). The highest values (in cm H₂O) from three repeated measurements for each parameter were analyzed.

Handgrip strength (HGS) was assessed with the use of the Jamar hand dynamometer (Saehan DHD-1, Masan, South Korea) according to the guidelines of the American Society of Exercise Physiologists (21). Three measurements were performed alternately for the left and right hands, with a minimum one-minute pause between attempts, and the highest value of generated strength was noted.

To assess quality of life, the 36-Item Short Form Health Survey (SF-36) and the Fatigue Severity Scale (FSS) were used.

### Intervention stage

#### Exercise training

A home-based exercise training and respiratory exercise program was tailored to each patient's clinical condition. Each participant received instruction on how to perform the exercises correctly, was informed about contraindications, principles of safe physical activity, and guidance on managing potential adverse effects during or after exercise. The participants and their caregiver(s) or family member(s), responsible for supervising the training, were instructed during an individual session. Each subject was provided with a booklet containing photographs and detailed descriptions of the exercises.

Each patient in the intervention group was provided with a Pulmogain RespiTrain device (Moretti, Italy) for resistance training of the respiratory muscles. Physical training, with intensity set at 4–5 points (fairly hard to hard) on the modified 10-point Borg Scale, was performed once daily, at least five times per week, for six months (24 weeks). Patients were advised to reach an optimal single-session duration of 45–60 min (minimum 30 min) within the first two weeks of starting the program. The rehabilitation program has been described in detail in previous publications (16, 22). A detailed, ready-to-use home-based physical training program (a brochure with photographs and comprehensive exercise descriptions) is provided as supplementary material (Brochure S1).

#### Self-monitoring and adherence

All training participants received pedometers (Pearly 3D, Spokey) to monitor daily physical activity and self-control diaries (Supplementary Table S1 in supplementary materials) to record data on physical activity parameters such as heart rate, blood pressure, fatigue, and dyspnoea using the 10-point Borg Scale before and after each training session. Additionally, the duration of each training session, the number of steps taken during the session, and the total daily steps (on both training and non-training days) were recorded.

Participants in the intervention group had scheduled visits one month and three months after starting the improvement program to monitor diary completion, verify exercise correctness, and make any necessary adjustments to the training program. Additionally, patients received telephone support at least once a week to verify adherence to protocol.

Adherence during the six-month intervention period was defined as the percentage of training sessions completed by patients per week, with a minimum of 120 sessions required over the 24-week period.

### Statistical analysis

Data analysis was performed using statistical environment R 3.6.2. Welch's ANOVA was performed for comparisons of multiple independent means (intergroup comparisons). The Kruskall-Wallis test was used as the non-parametric equivalent of Welch's ANOVA. For comparisons of the effects of the intervention in the study group a one-way analysis of variance for repeated measures was used. The Friedmann test was used as the nonparametric equivalent of this analysis.

Strength of effect for analyzes of variance was measured using the eta squared. The Bonferroni correction was used for *post-hoc* analysis. *P*-values < 0.05 were considered statistically significant. Data in the text are presented as mean ± standard deviation (SD).

## Results

Due to objective problems caused by the COVID-19 pandemic outbreak, the course and final outcomes of the study differed from the original assumptions. Specifically, assessment of the control group after six months was not feasible, as the majority were reluctant to attend regularly scheduled visits during this period—particularly because they did not receive any intervention. Consequently, we omitted the intergroup analysis following the rehabilitation program.

A total of 63 patients with CHF were enrolled in the study, of whom 28 patients from the intervention group completed the study and were included in the analysis. Patients were randomized in a 3:2 ratio, with 37 allocated to the intervention group and 26 to the control group. After post-randomization exclusions and dropouts, 28 and 26 patients remained in the respective groups, resulting in approximately balanced groups.

Figure 1 illustrates the flow of participants through the study.

**Figure 1:**
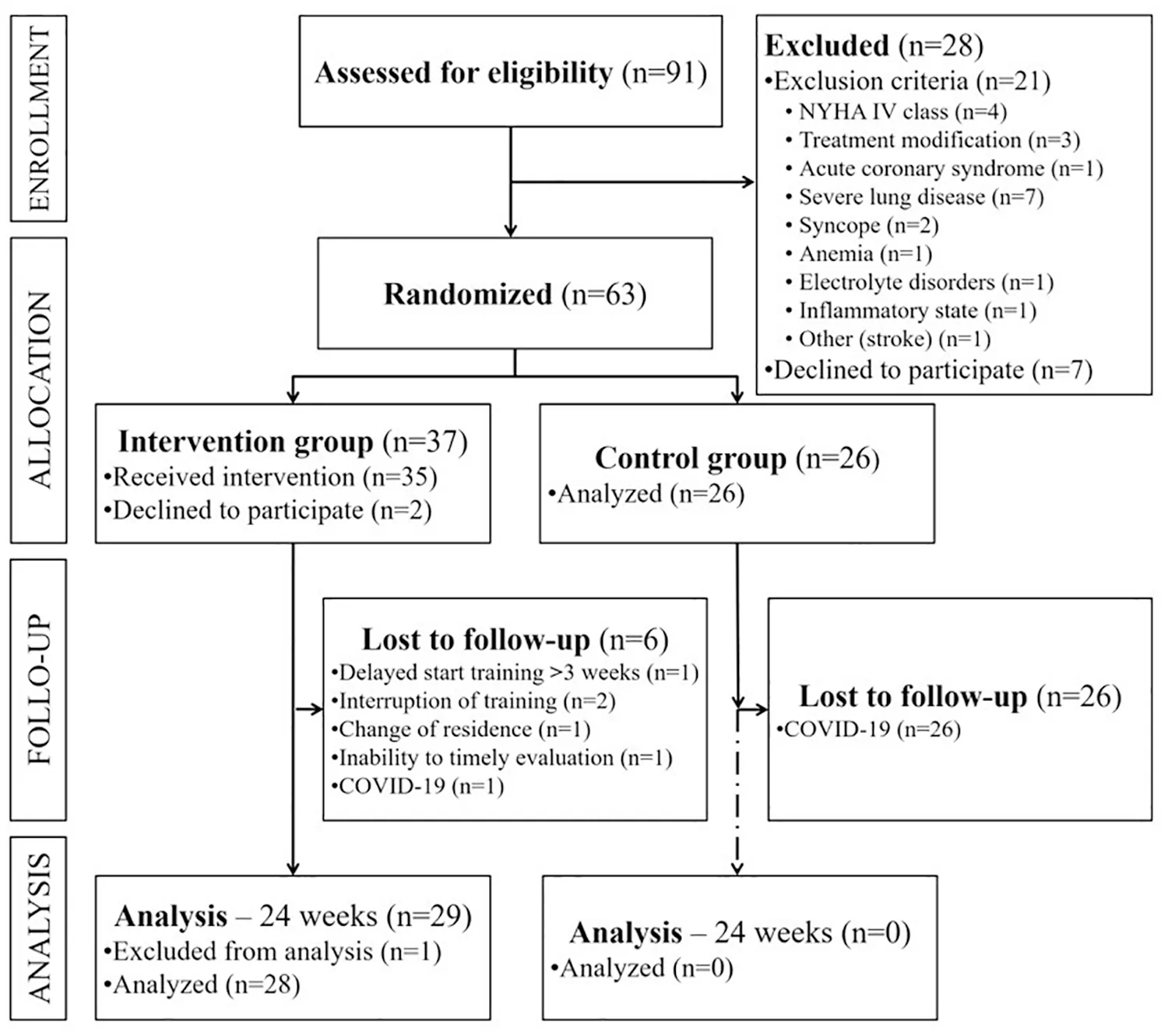
Study flowchart.

### Intervention group at baseline

Baseline characteristics of the intervention group are presented in Table 1.

**Table 1:** Baseline participants characteristics of intervention group.

Patients	28
Age (SD)	62.8 (8.1)
Male sex, *n* (%)	26 (93)
Weight, kg (SD)	90.75 (15.82)
Height, cm (SD)	174 (5.86)
Body Mass Index, kg/m^2^ (SD)	29.86 (4.28)
NYHA class	
II, *n* (%)	18 [64)
III, *n* (%)	10 (36)
Co-morbidities	
Coronary artery disease, *n* (%)	17 (61)
Arterial hypertension, *n* (%)	18 (64)
Atrial fibrillation, *n* (%)	11 (39)
Diabetes mellitus, *n* (%)	11 (39)
Hyperlipidemia, *n* (%)	21 (75)
Mitral regurgitation, *n* (%)	10 (36)
Medication	
ACEI, n(%)	24 (85.7)
Sartan, *n* (%)	3 (10.7)
Beta-blocker, *n* (%)	28 (100)
MRA, *n* (%)	27 (96.4)
Sacubitril/valsartan, *n* (%)	1 (3.6)
Furosemide, *n* (%)	12 (42.8)
Torasemide, *n* (%)	17 (60.7)
Loop diuretic, *n* (%)	25 (89.2)
Thiazide, *n* (%)	1 (3.6)
Ivabradine, *n* (%)	2 (7.1)
Digoxin, *n* (%)	3 (10.7)
ICD, *n* (%)	20 (71)
Echocardiography	
LVEF, % (SD)	27.14 (6.87)
Functional parameters	
6MWD, m (SD)	440.36 (62.94)
Dominant hand grip strength, kg (SD)	44 (9.23)
Non-dominant hand grip strength, kg (SD)	41.59 (8.63)
PImax, cmH_2_O (SD)	63.11 (20.29)
PEmax, cmH_2_O (SD)	103.32 (27.56)
SF-36	
Physical Component Summary (SD)	43.73 (6.12)
Mental Component Summary (SD)	44.87 (8.32)
Fatigue Severity Scale (SD)	4.01 (1.38)

Values are mean (standard deviation); 6MWD, 6-min walk distance; 95% CI, 95% confidence interval; ACEI, angiotensin converting enzyme inhibitor; ICD, implanted; cardioverter-defibrillator; LVEF, left ventricle ejection fraction; M diff, mean difference; MRA, mineralocorticoid receptor antagonist; NYHA, New York Health Association classification; PEmax, maximal expiratory pressure; PImax, maximal inspiratory pressure; SF-36, 36-Item Short Form Health Survey.

The mean age of patients in the intervention group was 62.8 ± 8.1 years, and most participants were men (93%). The mean BMI was 28.86 ± 4.38 kg/m^2^, indicating that the group was, on average, overweight. Most patients (64%) were classified as NYHA functional class II, and 61% had coronary artery disease. An implantable cardioverter-defibrillator was present in 71% of patients. The mean left ventricular ejection fraction was 27.14 ± 6.87%, while the mean 6-minute walk distance was 440.36 ± 62.94 m. The mean PImax and PEmax​ values were 63.11 ± 20.29 and 103.32 ± 27.56 cmH₂O, respectively. Mean handgrip strength was 44.00 ± 9.23 kg for the dominant hand and 41.59 ± 8.63 kg for the non-dominant hand.

### Training effects in the intervention group

*Among the 35 patients who initiated the home-based rehabilitation program, 29 completed the 24-week assessment, corresponding to a completion rate of 82.8%.*

A statistically significant improvement in the primary outcome, 6MWD, of 37.86 m was achieved after 6 months of a home-based rehabilitation program (*p* = 0.001, *η*^2^ = 0.1).

A 6-month exercise training program resulted in a statistically significant increase in respiratory muscle strength, as measured by PImax [83.32 (20.00) cmH₂O, *p* < 0.001, *η*^2^ = 0.16] and PEmax [124.07 (31.09) cmH₂O, *p* < 0.001, *η*^2^ = 0.11]. There was also a statistically significant improvement in the FSS (−0.68, *p* = 0.029, *η*^2^ = 0.06). *No statistically significant improvements were observed in quality of life, as assessed using the SF-36 questionnaire.*

Results of training effects are presented in Table 2. Post-intervention changes in 6MWD, PImax, and PEmax are shown in Figures 2–4, respectively.

**Table 2:** Training effects.

Functional parameters	Baseline (t1)	time 1→2		Postintervention (t2)	*η* ^2^
		change	*p*-value		
6MWD, m	440.36 ± 62.94	37.86	0.001	478.21 ± 78.16	0.1
PImax, cmH_2_O	63.11 (20.29)	20.21	<0.001	83.32 (20.3)	0.16
PEmax, cmH_2_O	103.32 (27.56)	20.75	<0.001	124.07 (31.09)	0.11
SaO_2_, % before 6MWT	96.21 (2.04)	0.61	0.111	96.82 (1.39)	0.04
SaO_2_, % after 6MWT	94.5 (2.62)	0.21	0.682	94.71 (2.16)	0
Dyspnea before 6MWT	0.86 (1.32)	−0.2	0.416	0.66 (1.05)	0.03
Dyspnea after 6MWT	4.71 (1.88)	−0.36	0.358	4.36 (2)	0.03
Fatigue before 6MWT	0.93 (1.27)	−0.2	0.404	0.73 (1.04)	0.02
Fatigue after 6MWT	4.86 (1.92)	−0.25	0.534	4.61 (2.11)	0.01
Dominant hand grip strength, kg	44 (9.23)	0.5	0.606	44.5 (9.26)	0.01
Nondominant hand grip strength, kg	41.49 (8.63)	1.15	0.241	42.74 (8.69)	0.01
SF-36					
Physical Functioning	60 (15.09)	3.21	0.192	63.22 (18.37)	0.03
Role Physical	49.11 (19.6)	3.12	0.378	52.23 (22.32)	0.03
Bodily Pain	71.29 (20.88)	-6.61	0.121	64.68 (22.74)	0
General Health	48.5 (13.63)	4.43	0.15	52.93 (16.51)	0.02
Vitality	54.46 (17.08)	0.22	0.945	54.69 (19.07)	0.01
Social Functioning	64.29 (23.25)	4.02	0.272	68.3 (21.91)	0.01
Role Emotional	62.2 (24.9)	2.68	0.602	64.88 (26.58)	0.02
Mental Health	65.36 (13.05)	−2.14	0.383	63.21 (16.79)	0
Physical Component Summary	43.73 (6.12)	0.62	0.46	44.34 (5.82)	0.02
Mental Component Summary	44.87 (8.32)	0.2	0.886	45.08 (10.04)	0
Fatigue Severity Scale	4.01 (1.38)	−0.68	0.029	3.33 (1.35)	0.06

Values are mean (standard deviation). 6MWD, 6-min walk distance; 6MWT, 6-min walk test; NS, not significant; PEmax, maximal expiratory pressure; PImax, maximal inspiratory pressure; SaO_2,_ oxygen saturation; SF-36, 36-Item short form health survey; time 1, measurements before intervention period; time 2, measurements after intervention period.

**Figure 2:**
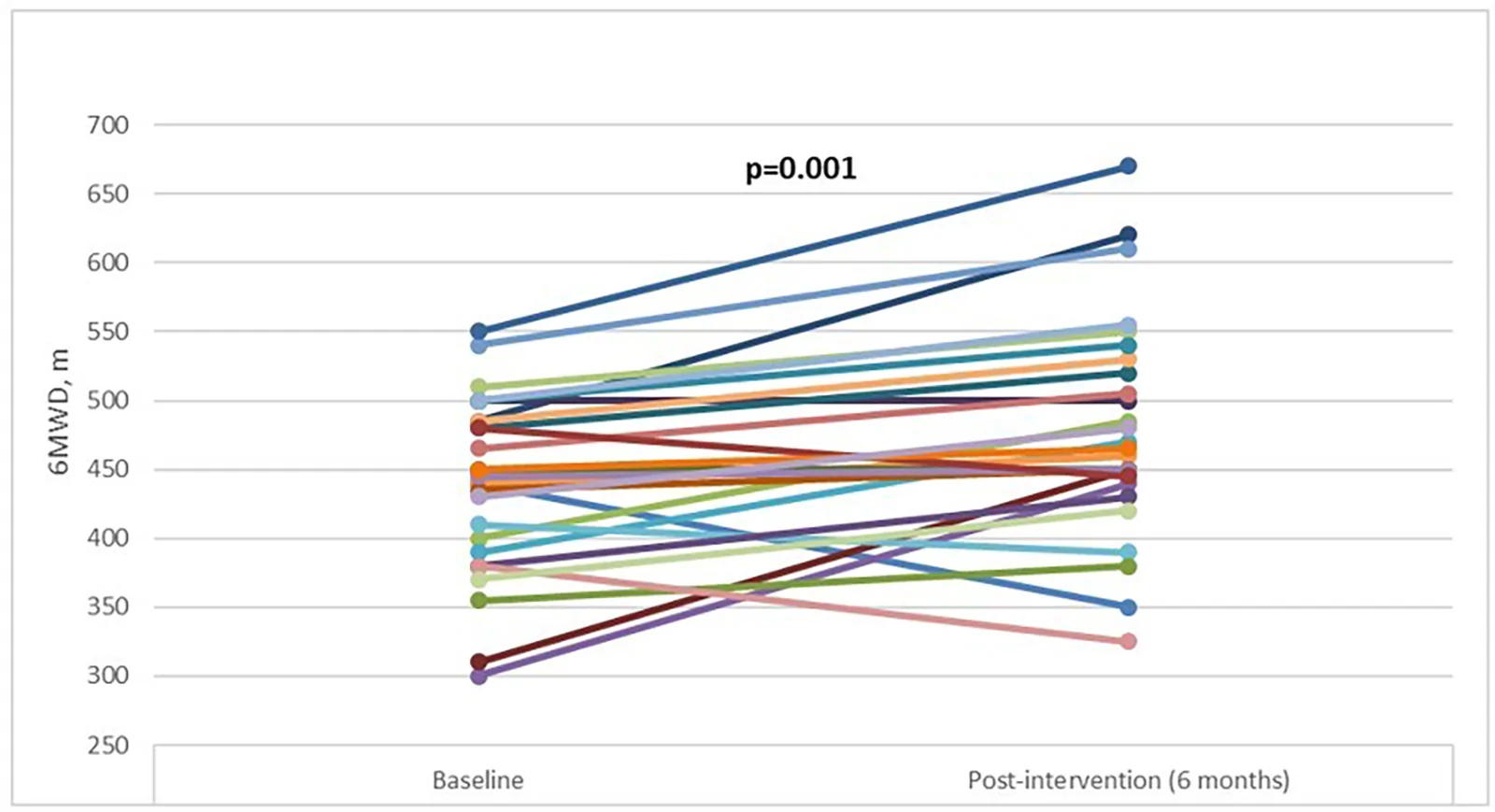
Post-intervention changes in 6MWD.

**Figure 3:**
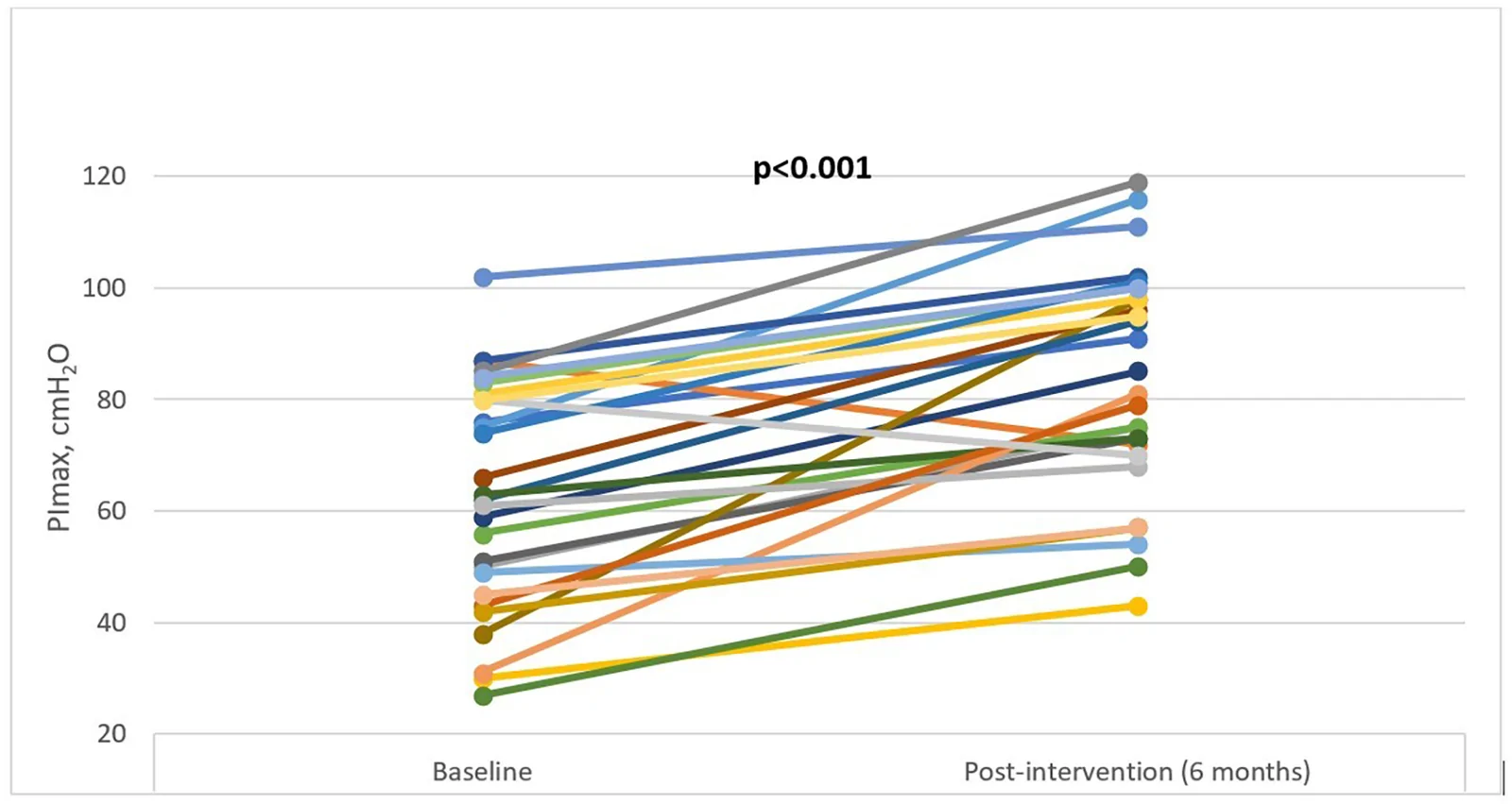
Post-intervention changes in PImax.

**Figure 4:**
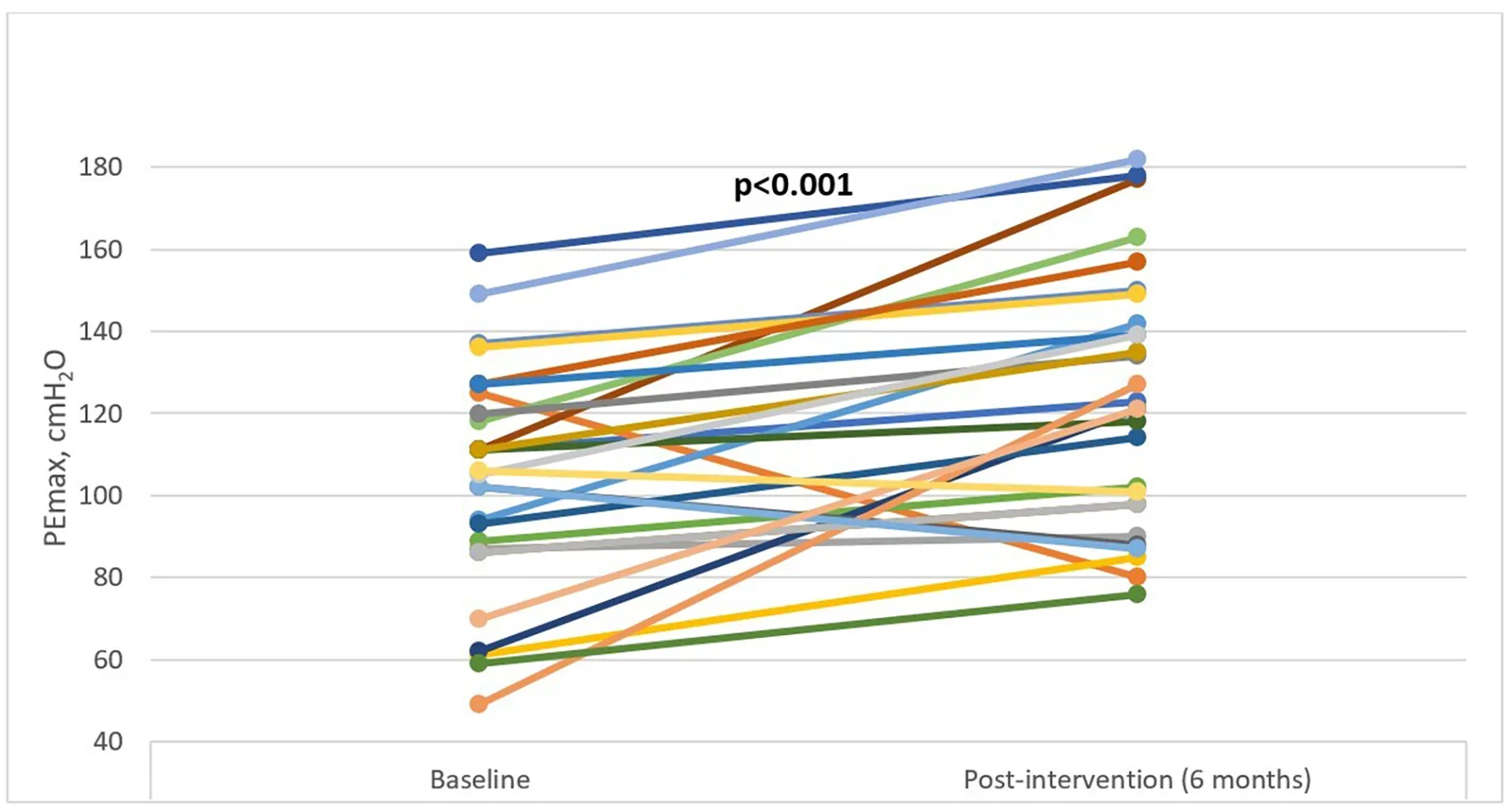
Post-intervention changes in PEmax.

Adherence to the recommended training protocol (at least 120 sessions in 24 weeks) was on average 91.5 (15.3) %. The total number of steps taken daily was 9,075 (3121), with an average number of steps taken during a single exercise session of 3,294 (1716), and an average daily number of steps in addition to training of 5,781 (2882). The average number of exercise sessions per week was 5.4 (1.4).

No statistically significant differences were noted between the mean total number of steps taken daily in the first and last weeks of the rehabilitation program [mean (SD) at week 1: 8901 (3560) vs. at week 24: 9234 (3632)], and the mean number of steps taken during a single training session [at week 1: 3192 (1656) vs. at week 24: 3254 (1857)].

Adverse events related to training were rarely reported and included excessive fatigue (*n* = 1), dyspnea (*n* = 1), chest pain (*n* = 2), and calf cramps (*n* = 1). There were no cases of death in the CHF group. Non-exercise-related adverse events included gastrointestinal complaints (*n* = 2), increased sweating (*n* = 1), common cold (*n* = 1), pneumonia (*n* = 1), and pulmonary edema (*n* = 1).

## Discussion

This pilot study over a 6-month period demonstrates the effectiveness and safety of a simple, low-cost home-based training program for patients with CHF. Because a control-group assessment was not available at 6 months, the observed improvement cannot be attributed exclusively to the intervention. Part of the change may reflect natural variability, regression to the mean, or nonspecific effects, including increased attention, participant expectations, and placebo-related effects. Therefore, these findings should be interpreted as evidence of improvement over time rather than definitive proof of an intervention-specific effect.

It is believed that cardiac rehabilitation is underused, with participation rates ranging from 10% to 30% (23–25). In HF-ACTION clinical trial, conducted on a group of 2,331 patients with HFrEF (EF <35%), participants were randomized to 36 sessions of supervised exercise training over 12 weeks, followed by up to 1 year of home-based exercise (5 sessions per week) (26). Despite intensive efforts to increase adherence, long-term adherence was less than 30% (4, 26). In the present study, patients’ adherence to the recommended home-based training protocol, which included a minimum of 120 sessions over 24 weeks, was on average 91.5%.

A systematic review including a total of 23 trials that randomized 2,890 participants after acute myocardial infarction, revascularization, or with HF, compared the effects of home-based and supervised center-based cardiac rehabilitation (27). No statistically significant differences were observed between home- and center-based cardiac rehabilitation in clinical outcomes up to 12 months of follow-up, including total mortality [relative risk (RR) = 1.19] and exercise capacity (standardized mean difference = −0.13). Marginally higher program completion rates (RR = 1.04) were observed among home-based participants. In our study, completion of the home-based rehabilitation program was high, at 82.8%.

In the study by Cowie et al., which focused on a group of patients with HF and compared the effects of home-based versus center-based rehabilitation, the completion rates for 16 exercise sessions over 3 months were 77% and 86%, respectively (28).

Our data on the high completion rate are consistent with findings from the meta-analysis by Zwisler et al. (15), which showed that home-based cardiac rehabilitation for CHF patients was associated with higher trial completion rates compared to center-based rehabilitation (relative risk: 1.2; 95% CI: 1.0 to 1.3).

Typical comprehensive cardiac rehabilitation programs are based on physical training. Some studies emphasize the role of breathing exercises and IMT in improving exercise capacity, functional performance, as well as ventilation and ventilation-to-perfusion matching (11). However, the optimal mode of IMT and the characteristics of patients who are most likely to benefit from this type of intervention remain unclear.

In a meta-analysis, Azambuja et al. analyzed the impact of IMT on functional capacity, respiratory muscle strength, quality of life, and dyspnoea in HF patients with. Of the 13 included studies, 10 focused on isolated IMT, while 3 investigated IMT combined with another intervention (12).

Isolated inspiratory muscle training resulted in increases in PImax by 25.12 cm H₂O, 6MWD by 81.18 meters, and maximum oxygen consumption after 12 weeks by 3.75 mL/kg/min. Greater improvements in PImax were observed in patients with inspiratory muscle weakness, higher training loads, and longer intervention durations. In contrast, IMT combined with another intervention showed an increase only in PImax.

In the present study, respiratory muscle strength in the intervention group was 63.11 (20.29) cmH₂O for PImax, and a statistically significant increase in PImax of 20.21 cmH₂O was observed (*p* < 0.001), along with a clinically meaningful improvement in 6MWD of 37.86 meters (*p* = 0.001). These findings are consistent with data from meta-analyses indicating that patients with inspiratory muscle weakness experience greater increases in PImax.

Neto et al. reviewed the impact of combining exercise and IMT on the rehabilitation outcomes of patients with HF (13). Cardiac rehabilitation based on this combined approach resulted in an improvement in PImax of 20.89 cmH₂O, which is very close to the improvement observed in the present study, where PImax increased by 20.21 cmH₂O.

A meta-analysis of eight studies involving 302 patients with CHF evaluated the positive effect of IMT on respiratory muscle strength (14). IMT significantly improved PImax by 16.52 cmH₂O.

### Study limitations

An important limitation of this study is that, because no control-group assessment was available after 6 months, the primary longitudinal comparison effectively constituted a single-arm pre–post analysis. Consequently, the observed changes cannot be attributed exclusively to the intervention, and the durability of the effects cannot be reliably compared with that of a control group. In addition, the relatively small sample size limits the statistical power and generalizability of the findings.

Adherence monitoring was based on patient self-reports, supervised by a caregiver or family member, and weekly telephone verification by the study team. Modern approaches to rehabilitation supervision increasingly rely on telemonitoring, including the use of implantable cardiac devices (29). However, in times of economic crisis, simple and cost-effective methods—such as self-reports supported by accelerometers and telephone calls—can serve as a viable alternative, especially in low-resource settings.

Another limitation was caused by the COVID-19 pandemic, which made it impossible to assess the control group six months after patient inclusion. The inability to compare the functional status of patients in the CHF intervention group after six months of the improvement program with those who did not undergo training limits the capacity to determine the extent of disease-related physical impairment over time. Due to the pandemic and the introduction of lockdown measures, control group patients were reluctant to participate in the final evaluation. Considering the benefit-risk ratio and the increased risk of severe COVID-19 infection, no insistence was made on the control group's participation in the follow-up assessment.

Another important issue concerns the medical treatment of the CHF patient: no flozins were used, and only a small percentage of patients received sacubitril/valsartan (3.6% in the intervention group). This study began before the widespread adoption of these drugs in clinical practice, and efforts were made to maintain comparable treatment regimens among CHF patients.

## Conclusions

The present research provides key data on the feasibility of the developed home-based exercise improvement program, demonstrating it as an acceptable, safe, and effective alternative for CHF patients who lack access to other forms of rehabilitation. Home-based cardiac rehabilitation may be particularly valuable during the pandemic period, as it allows patients to avoid gathering in a single center simultaneously. Despite the challenges posed by conducting the study during the COVID-19 pandemic, the proven positive effects of home-based rehabilitation underscore the usefulness of this approach even in extraordinary circumstances such as a pandemic.

## Data Availability

The raw data supporting the conclusions of this article will be made available by the authors, without undue reservation.
