# Supplementary material for: Beyond the clinic: a six-month home-based exercise and respiratory rehabilitation program improves functional capacity, respiratory muscle strength, and fatigue in chronic heart failure
Source: Front Rehabil Sci. 2026 Oct 14;7:1958027. doi: 10.3389/fresc.2026.1958027 (PMC13713856; doi:10.3389/fresc.2026.1958027)
Supplement: Supplementary file 2 [file Table1.doc]

| **DATE** | **BEFORE TRAINING** | | | | | | **AFTER TRAINING** | | | | | |
| --- | --- | --- | --- | --- | --- | --- | --- | --- | --- | --- | --- | --- |
|  | dyspnea &fatigue (scale 0–10) | blood pressure | heart rate | blood oxygen saturation | number of steps | start time | finish time | dyspnea &fatigue (scale 0–10) | blood pressure | heart rate | blood oxygen saturation | number of steps |
|  |  |  |  |  |  |  |  |  |  |  |  |  |
|  |  |  |  |  |  |  |  |  |  |  |  |  |
|  |  |  |  |  |  |  |  |  |  |  |  |  |
|  |  |  |  |  |  |  |  |  |  |  |  |  |
|  |  |  |  |  |  |  |  |  |  |  |  |  |
|  |  |  |  |  |  |  |  |  |  |  |  |  |
|  |  |  |  |  |  |  |  |  |  |  |  |  |
|  |  |  |  |  |  |  |  |  |  |  |  |  |
|  |  |  |  |  |  |  |  |  |  |  |  |  |
|  |  |  |  |  |  |  |  |  |  |  |  |  |
|  |  |  |  |  |  |  |  |  |  |  |  |  |
|  |  |  |  |  |  |  |  |  |  |  |  |  |
|  |  |  |  |  |  |  |  |  |  |  |  |  |
|  |  |  |  |  |  |  |  |  |  |  |  |  |
|  |  |  |  |  |  |  |  |  |  |  |  |  |
